# Novel MenA Inhibitors Are Bactericidal against *Mycobacterium tuberculosis* and Synergize with Electron Transport Chain Inhibitors

**DOI:** 10.1128/AAC.02661-18

**Published:** 2019-05-23

**Authors:** Bryan J. Berube, Dara Russell, Lina Castro, Seoung-ryoung Choi, Prabagaran Narayanasamy, Tanya Parish

**Affiliations:** a TB Discovery Research, Infectious Disease Research Institute, Seattle, Washington, USA; b Department of Pathology, University of Nebraska Medical Center, Omaha, Nebraska, USA; c Department of Microbiology, University of Nebraska Medical Center, Omaha, Nebraska, USA

**Keywords:** *Mycobacterium tuberculosis*, antitubercular, bactericidal, electron transport chain, menaquinone, respiration, synergy, tuberculosis

## Abstract

Mycobacterium tuberculosis is the leading cause of morbidity and death resulting from infectious disease worldwide. The incredible disease burden, combined with the long course of drug treatment and an increasing incidence of antimicrobial resistance among M. tuberculosis isolates, necessitates novel drugs and drug targets for treatment of this deadly pathogen. Recent work has produced several promising clinical candidates targeting components of the electron transport chain (ETC) of M. tuberculosis, highlighting this pathway’s potential as a drug target. Menaquinone is an essential component of the M. tuberculosis ETC, as it functions to shuttle electrons through the ETC to produce the electrochemical gradient required for ATP production for the cell. We show that inhibitors of MenA, a component of the menaquinone biosynthetic pathway, are highly active against M. tuberculosis. MenA inhibitors are bactericidal against M. tuberculosis under both replicating and nonreplicating conditions, with 10-fold higher bactericidal activity against nutrient-starved bacteria than against replicating cultures. MenA inhibitors have enhanced activity in combination with bedaquiline, clofazimine, and inhibitors of QcrB, a component of the cytochrome *bc*_1_ oxidase. Together, these data support MenA as a viable target for drug treatment against M. tuberculosis. MenA inhibitors not only kill M. tuberculosis in a variety of physiological states but also show enhanced activity in combination with ETC inhibitors in various stages of clinical trial testing.

## Abstract (precis)

Mycobacterium tuberculosis is the leading cause of morbidity and death resulting from infectious disease worldwide. The incredible disease burden, combined with the long course of drug treatment and an increasing incidence of antimicrobial resistance among M. tuberculosis isolates, necessitates novel drugs and drug targets for treatment of this deadly pathogen.

## INTRODUCTION

Mycobacterium tuberculosis, the causative agent of tuberculosis, is a major burden on global public health systems, infecting ∼2 billion people, with more than 10 million new cases of active disease in 2017 (1, 2). While most of these cases are characterized as “latent” infections, it is estimated that up to 10% of patients progress to active disease during their lifetimes. The long course of drug treatment, lack of public health infrastructure, and increases in antibiotic resistance have led to M. tuberculosis becoming the leading cause of death from infectious disease in the world, with 1.3 million attributable deaths in 2017 (1). These numbers, combined with the increasing rates of multidrug-resistant and extensively drug-resistant strains, have led to renewed efforts to find both novel compounds active against M. tuberculosis and novel targets to attack as part of a multidrug regimen that can escape bacterial resistance.

The mycobacterial electron transport chain (ETC) has garnered significant interest as a drug target. M. tuberculosis is an obligate aerobe that uses oxidative phosphorylation for ATP production to fuel cellular processes (3, 4). During oxidative phosphorylation, electrons flow through the ETC from membrane dehydrogenases through a quinone intermediate to terminal oxidases. Electron flow is coupled to the establishment of a proton gradient, which is used by the F_1_F_0_ ATPase to synthesize ATP (3, 4). ATP production is critical for the viability of M. tuberculosis during active disease and also for the maintenance of basal metabolic activity during latent infection (5, 6).

New drugs that target components of the ETC and ATP production have been discovered. Bedaquiline (BDQ), which directly targets the F_1_F_0_ ATPase, is the first tuberculosis drug approved by the FDA for limited use in 40 years (7–9). BDQ works as an uncoupler, allowing proton flow through the ATPase without the benefit of ATP production, thereby depleting cells of ATP (8). Clofazimine (CLO) acts partly by targeting NADH dehydrogenase and kills M. tuberculosis cells through the production of reactive oxygen species (10, 11). In addition, a number of compounds that target QcrB, a component of the cytochrome *bc*_1_-*aa*_3_ terminal oxidase, have been identified (12–17). Among these, the imidazopyridine Q203 is the most advanced in phase II clinical trials. QcrB inhibition has several effects on M. tuberculosis, including depletion of intracellular ATP and disruption of pH homeostasis (12, 14–16). The success of these compounds highlights the viability of targeting the ETC as a way to treat M. tuberculosis during any state of infection.

In M. tuberculosis, menaquinone is a central and critical component of the ETC; it is the predominant quinone found in mycobacteria, serving as an electron shuttle to the terminal reductases (18). Menaquinone is synthesized from chorismate by a series of eight enzymes (MenF, MenD, MenH, MenC, MenE, MenB, MenA, and MenG), most of which are considered essential for growth (4, 19, 20). Because humans acquire menaquinone through their diet, these enzymes are not present in human cells and therefore are attractive as selective drug targets. To date, chemical inhibitors of MenA (20), MenB (21), MenG (22), and MenE (23) have proven efficacious in inhibiting M. tuberculosis growth, validating the essentiality of this pathway. In this study, we characterize the activity of MenA inhibitors against M. tuberculosis. MenA inhibitors not only prevent M. tuberculosis growth but also are bactericidal and have synergistic activity in combination with compounds targeting other components of the ETC. This work validates MenA as a viable target in the treatment of M. tuberculosis and highlights its potential for use in a novel drug regimen targeting the ETC.

## RESULTS

**FIG 1:**
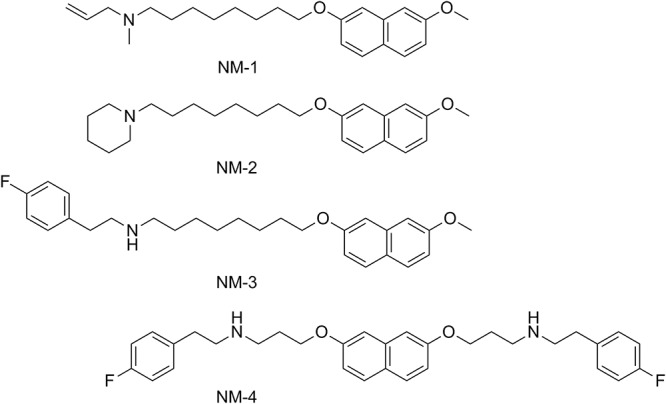
Structures of MenA inhibitors used in this study.

Previous work identified novel inhibitors of MenA that were active against numerous bacteria, including nontuberculous mycobacteria (24) (Fig. 1). On-target activity of these compounds is suggested by growth inhibition of Staphylococcus aureus being rescued by supplementation with menaquinone (MK-4) and by the compounds directly inhibiting M. tuberculosis MenA enzyme activity (24, 25), although the possibility that whole-cell activity results from inhibition of additional targets cannot be excluded. Given the need for novel antibacterials to treat M. tuberculosis and the essentiality of menaquinone to the bacterium’s survival, we tested the MenA inhibitors against whole-cell M. tuberculosis H37Rv-LP. MenA-targeting compounds inhibited the growth of M. tuberculosis, and NM-4 was the most potent, with a MIC of 4.5 μM (Table 1).

**TABLE 1:** Activity of MenA inhibitors against M. tuberculosis

Compound	MIC (μM)^*a*^		
	H37RvLP	H37RvMA	H37RvMA Δ*cydC*::*aph*
NM-1	41 ± 2	55 ± 4	38 ± 3
NM-2	42 ± 2	49 ± 2	43 ± 4
NM-3	14 ± 0.2	15 ± 0.2	15 ± 2
NM-4	4.5 ± 0.7	5.5 ± 1.7	3.8 ± 0.2

a MenA inhibitors were tested against M. tuberculosis. MICs were calculated as the minimum concentrations required to inhibit the growth of M. tuberculosis by 90%, as determined by Levenberg-Marquardt least-squares plots. Data are the mean ± standard deviation of two independent experiments.

Many ETC inhibitors suffer from redundancies in the respiratory pathway encoded in the genome of M. tuberculosis. When challenged with select ETC inhibitors, M. tuberculosis is capable of respiratory flexibility that decreases the effectiveness of the compounds. One major route of respiratory flexibility involves upregulation of the alternative terminal electron acceptor cytochrome *bd*, which provides resistance to numerous inhibitors of the ETC (26–32). Importantly, knockout of the cytochrome *bd* oxidase in M. tuberculosis did not increase susceptibility to the MenA inhibitors (Table 1), indicating that this prominent escape route does not provide resistance to NM1-4.

**FIG 2:**
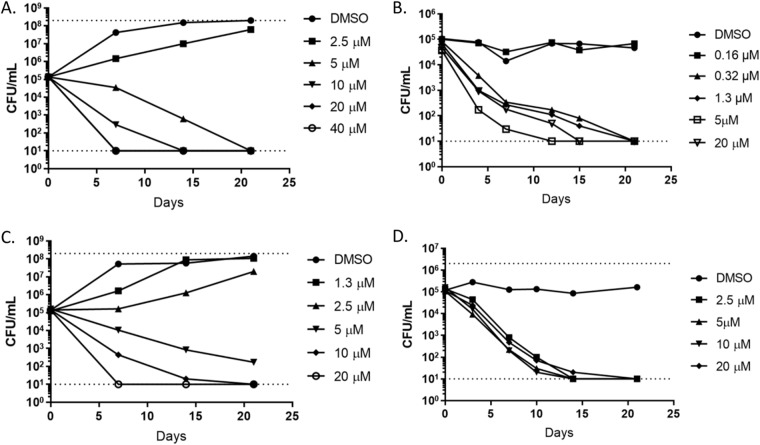
MenA inhibitors are bactericidal against M. tuberculosis. M. tuberculosis H37RvLP was cultured in the presence of the indicated concentration of NM-4 under aerobic (individual replicates in A and C) or starvation (individual replicates in B and D) conditions. Samples were taken at the indicated times. The dotted lines represent the upper and lower limits of detection.

Because NM-4 was the most potent compound, we tested its ability to kill M. tuberculosis. Under aerobic growth conditions, NM-4 was bactericidal in a concentration-dependent manner (Fig. 2A and C); all concentrations above the MIC killed M. tuberculosis within 21 days. At 20 μM (∼5× MIC), NM-4 sterilized the culture rapidly, within 7 days (Fig. 2A). We next tested its ability to kill M. tuberculosis under nutrient starvation conditions, a physiological state that is likely to be highly relevant *in vivo* and in which M. tuberculosis is recalcitrant to many antibiotics (33). Surprisingly, NM-4 was even more active under nutrient-starved nonreplicating conditions than during aerobic growth. Concentrations as low as 0.32 μM sterilized the culture within 21 days (Fig. 2B and D), which represented a 10-fold increase in potency, compared to bactericidal concentrations under aerobic conditions.

**FIG 3:**
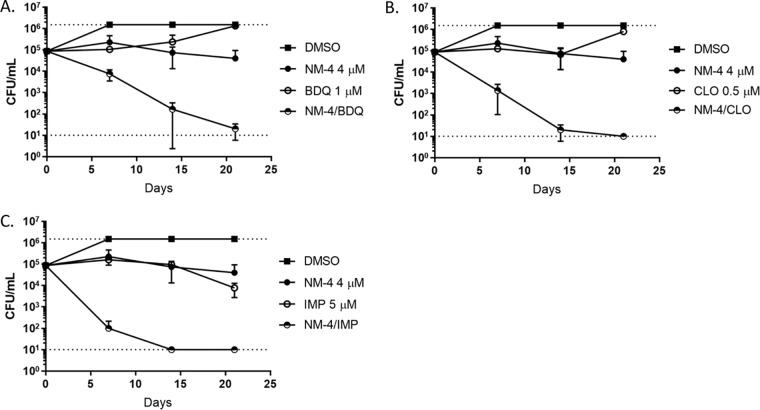
NM-4 causes synergistic killing with inhibitors of the ETC. Killing kinetics of NM-4 at approximately 1× MIC in combination with BDQ (A), CLO (B), or IMP (C) at subbactericidal concentrations were assessed under replicating conditions. Combinations were tested against H37RvLP. Data are the mean ± standard deviation of two independent experiments. The dotted lines represent the upper and lower limits of detection.

Because treatment of M. tuberculosis requires a multidrug regimen, we tested NM-4 in combination with several other inhibitors of the ETC under aerobic conditions. In order to see potential synergy of selected combinations, we used concentrations of inhibitors that were low enough to inhibit the growth of M. tuberculosis without causing substantial killing on their own. A low concentration of NM-4 caused synergistic killing in combination with subbactericidal concentrations of BDQ, CLO, and an imidazopyridine (IMP) compound (ND-10885 [34]) (Fig. 3). All combinations of drugs sterilized M. tuberculosis cultures within 21 days. The NM-4-IMP combination was the most potent, causing nearly complete sterilization of the culture within only 7 days, similar to a concentration of 20 μM NM-4 on its own (Fig. 2A), i.e., a 5-fold enhancement of potency.

## DISCUSSION

Our data provide strong evidence supporting menaquinone synthesis as a viable and attractive drug target. Compounds targeting MenA not only inhibit the growth of M. tuberculosis but also have potent bactericidal activity, particularly under nutrient starvation conditions. As M. tuberculosis infection progresses *in vivo*, bacteria reside within granulomas characterized by nutrient-poor and/or oxygen-poor conditions (35). In these nonreplicating states, the flow of electrons through the ETC is critical for M. tuberculosis to maintain both membrane potential and the low-level ATP production required to keep basal cellular processes active (6, 36). The ability of NM-4 to kill M. tuberculosis 10-fold more effectively under nutrient starvation conditions suggests that menaquinone biosynthesis inhibitors could be highly efficient in killing both replicating and “latent” bacteria. The ability to target both populations is particularly attractive in a drug candidate. Many of the current frontline drugs are active only against replicating bacteria; therefore, a drug targeting both populations is predicted to significantly shorten treatment time (37).

With the recent successes of BDQ, CLO, and Q203, the ETC has received significant attention in the development of novel drug regimens to treat M. tuberculosis infections. These three compounds have all proven efficacious *in vitro* and *in vivo*, particularly in combination treatments (7, 16, 27–29). Despite this, there is still a great deal of skepticism regarding the utility of targeting the ETC. Many components of the M. tuberculosis ETC have redundancies that allow for escape from chemical or genetic inhibition. The clearest example is the ability of M. tuberculosis to reroute electron flow to the alternative terminal oxidase cytochrome *bd* upon chemical or genetic inhibition of QcrB and the cytochrome *bc*_1_ complex (26–28, 38). The ability of M. tuberculosis to reroute electron flow through alternative components of the ETC could limit the use of some drugs as the sole component targeting the ETC in a new drug regimen.

However, the redundancy across different complexes of the ETC extends only so far. Genes encoding the F_1_F_0_ ATPase and the enzymes responsible for menaquinone biosynthesis are present in only a single copy, with no known functional homologues (4, 36). Despite this advantage, there is always the possibility of undiscovered alternative pathways for routing electrons through the ETC. In fact, an alternative polyketide quinone was recently discovered to be utilized under low-oxygen conditions (39). However, much of this work was done in Mycobacterium smegmatis, and it is still unclear how these findings translate to M. tuberculosis.

In order to combat potential rerouting of the ETC as well as the evolution of resistant mutants, menaquinone inhibitors should be given as part of a multidrug regimen. Our data highlight a major advantage of targeting the menaquinone pathway, i.e., MenA inhibitors synergize with all tested ETC inhibitors. Low doses of NM-4 acted synergistically with subbactericidal concentrations of BDQ, CLO, and an IMP, causing enhanced and efficient killing of M. tuberculosis. We hypothesize that NM-4 synergizes with other ETC inhibitors by decreasing the pool of menaquinone in the cell, thus limiting electron flow to complex III/IV and complex V of the ETC. Any further insult to complex III/IV (with QcrB inhibitors) or complex V (with BDQ) would severely disrupt production of ATP and render the bacterium unviable, although this needs to be shown experimentally. Whatever the mechanism, the synergistic activity of NM-4 with a range of ETC inhibitors opens the window to a number of different combination opportunities, which can be tailored based on the drug sensitivities of individual strains or different safety profiles.

Taken together, our data support the development of menaquinone inhibitors as the centerpiece of a novel drug regimen to treat M. tuberculosis. MenA inhibitors have good biological profiles, as described above, and should provide a good safety window, given that the enzyme is absent from humans. Pharmacokinetic and pharmacodynamic studies need to be carried out to provide a proof of concept for menaquinone inhibition in an animal model of disease.

## MATERIALS AND METHODS

### Bacterial strains and growth conditions.

The bacterial strains used in these studies were M. tuberculosis H37RvLP (ATCC 25618), H37RvMA (ATCC 27294), and H37RvMA Δ*cyd* (26) (provided by Helena Boshoff). All strains were grown under aerobic conditions in Middlebrook 7H9 medium containing 10% (vol/vol) oleic acid-albumin-dextrose-catalase (OADC) (Becton, Dickinson) and 0.05% (wt/vol) Tween 80 (7H9-Tw-OADC). When indicated, strains were nutrient starved by incubation for 2 weeks in phosphate-buffered saline (PBS) with 0.05% (wt/vol) tyloxapol.

### Determination of MICs.

MICs were determined as described previously (40); briefly, M. tuberculosis was grown under aerobic conditions in 96-well plates in 7H9-Tw-OADC. After 5 days of incubation at 37°C, bacterial growth was measured as the optical density at 590 nm (OD_590_). The MIC was defined as the concentration of compound required to inhibit the growth of M. tuberculosis by 90%, and values were determined using Levenberg-Marquardt least-squares plots.

### Determination of compound killing kinetics.

M. tuberculosis was inoculated at ∼2 × 10^5^ CFU/ml into 7H9-Tw-OADC containing compound (final dimethyl sulfoxide [DMSO] concentration of 2%). Standing cultures were incubated for 3 weeks at 37°C, and CFU were determined by plating serial dilutions. For starvation, M. tuberculosis was nutrient starved for 2 weeks prior to compound addition.
